# Editorial: Glycotherapeutics: Design, synthesis, function and biomedical application of agents emerging from glycochemistry and glycobiology

**DOI:** 10.3389/fmolb.2022.1012485

**Published:** 2022-09-08

**Authors:** M. Osman Sheikh, Chantelle J. Capicciotti, Stéphanie Olivier-Van Stichelen

**Affiliations:** ^1^ Discovery Science Division, Amicus Therapeutics, Inc., Philadelphia, PA, United States; ^2^ Departments of Chemistry, Biomedical and Molecular Sciences, and Surgery, Queen’s University, Kingston, ON, Canada; ^3^ Department of Biochemistry, Medical College of Wisconsin, Milwaukee, WI, United States

**Keywords:** glycans, glycomics, chemical biology, glycoengineering, carbohydrates

Post-translational glycosylation is a non-template driven process for the addition of carbohydrates, or glycans, to biomolecules such as protein and lipids. It is well recognized that glycosylation impacts many cellular processes including, but not limited to, protein folding, trafficking, receptor binding, signaling, inflammation, and cell-to-cell/matrix adhesion (Varki 2017). Additionally, glycans can be used in the targeting of drugs in diseases such as cancer (Diniz et al., 2022) and lysosomal storage disorders (Do et al., 2019) as well as modulating pharmacokinetics and immunogenicity of therapeutic proteins and antibodies (Liu 2015; Archer et al., 2022; Dammen-Brower et al., 2022).

The goal of this Research Topic was to assemble a collection of articles highlighting recent advancements pertaining to diagnostic tools and therapeutics utilizing multidisciplinary approaches in glycobiology and glycochemistry. Collectively, four articles submitted by 21 experts in glycoscience were peer-reviewed and accepted for publication in this Research Topic to emphasize the importance of glycans in human health and disease, in addition to presenting rapidly evolving technologies used in the analysis of carbohydrate-modified molecules and the enzymes that synthesize or degrade them.

A review article by Loaeza-Reyes et al. provides a comprehensive overview of protein *N*-linked and *O*-linked glycosylation and their implications in cardiovascular function and disease, including an analysis of cardiovascular disease risk factors associated with aberrant *N*-glycosylation and the dynamic role of the *O*-GlcNAc modification in inflammation. With more than 7,800 proteins modified in humans, *O*-GlcNAcylation is a critical modulator of signaling pathways in health and diseases (Wulff-Fuentes et al., 2021). Taking an analytical approach, Burt et al. provide a review of this dynamic modification and strategies to elucidate *O*-GlcNAc-modified proteins using high performance liquid chromatography coupled to tandem mass spectrometry (LC-MS/MS). The glycosidic bond between GlcNAc and serine or threonine residues is inherently labile, thus careful MS fragmentation strategies must be taken into consideration in order to accurately detect *O*-glycopeptides. While the mass spectrometer cannot discern the two HexNAc stereoisomers, GlcNAc and GalNAc, the authors summarize efforts in the literature to accurately identify the correct stereoisomers based on the ratio of fragment ions produced from the respective glycans. Additionally, Mukherjee et al. contributed a review article describing synthetic approaches to stereoselective chemical *O*-glycosylation reactions, which can be extremely challenging. This valuable summary of methods will aid the synthetic chemist in deriving a strategy for synthesizing target glycans with defined glycosidic linkages, including 1,2-*cis*, 1,2-*trans* and 2-deoxy-glycosides. Finally, a research article by Howlader et al. studied human neuraminidase enzymes and their roles in transmigration using an *in vitro* system. Here, the authors investigated the pharmacological inhibition of the NEU1, NEU3, and NEU4 isoenzymes. They propose these enzymes are positive regulators of transmigration and are potential targets for anti-inflammatory approaches.

In summary, within this Research Topic we sought to highlight the recent advancements in Glycoscience and how they can be utilized for therapeutic approaches to human diseases. We hope this collection of articles will be of interest to the broader scientific community and facilitate further discussion.

## References

[B1] Archer E. J. Gonzalez J. C. Ghosh D. Mellins E. D. Wang T. T. (2022). Harnessing IgG Fc glycosylation for clinical benefit. Curr. Opin. Immunol. 77, 102231. 10.1016/j.coi.2022.102231 35797920 PMC9870045

[B2] Dammen-Brower K. Epler P. Zhu S. Bernstein Z. J. Stabach P. R. Braddock D. T. (2022). Strategies for glycoengineering therapeutic proteins. Front. Chem. 10, 863118. 10.3389/fchem.2022.863118 35494652 PMC9043614

[B3] Diniz F. Coelho P. Duarte H. O. Sarmento B. Reis C. A. Gomes J. (2022). Glycans as Targets for Drug Delivery in Cancer, 14. Cancers (Basel). 10.3390/cancers14040911 PMC8870586 35205658

[B4] Do H. V. Khanna R. Gotschall R. (2019). Challenges in treating pompe disease: An industry perspective. Ann. Transl. Med. 7, 291. 10.21037/atm.2019.04.15 31392203 PMC6642928

[B5] Liu L. (2015). Antibody glycosylation and its impact on the pharmacokinetics and pharmacodynamics of monoclonal antibodies and Fc-fusion proteins. J. Pharm. Sci. 104, 1866–1884. 10.1002/jps.24444 25872915

[B6] Varki A. (2017). Biological roles of glycans. Glycobiology 27, 3–49. 10.1093/glycob/cww086 27558841 PMC5884436

[B7] Wulff-Fuentes E. Berendt R. R. Massman L. Danner L. Malard F. Vora J. (2021). The human O-GlcNAcome database and meta-analysis. Sci. Data 8, 25. 10.1038/s41597-021-00810-4 33479245 PMC7820439

